# Continuous Gum Dentures

**Published:** 1882-11

**Authors:** 


### EDITORIAL.

### Continuous Gum Dentures.

The greatest difficulty and embarrassment that has attended the construction of continuous gum dentures, have been found in the operation and management of the furnace or furnaces employed.

To all who have experience in this matter, a bare mention is sufficient, and to those who have not such experience, any description that could be given here would fail to give an adequate impression.

The difficulties that attend the furnace work has been one of the great obstacles to its more general introduction and use.

The furnaces in common use, up to within a recent period, involved much tedious and difficult manipulation. From six to ten hours were required for the furnace work alone, for the production of a continuous gum piece, and from two to five bushels of coke or antharcite coal were required for an operation, and a workman of salamander quality to run the machine. But now how changed—there is some risk in stating the whole truth on this subject, lest ones reputation for veracity suffers. Dr. A. Tees' Lilliput furnace for continuous gum work has wrought a revolution, or at least a remarkable improvement.

In the first place the size of the furnace is reduced to fifteen inches high, twelve inches wide and eight inches deep. Its muffle is large enough for any continuous gum denture.

It is constructed in three sections, so that it is easy to keep it perfectly clean; it is the work of but a few moments to charge the furnace for a full heat; twenty-five to thirty-five minutes from the moment of lighting the fire is required for bringing up the heat to the fusing point of the porcelain. It is uniform in its operation, so rapid is the running up of the heat that the outside of the furnace does not become hot during the time required for one operation, and at any time the operator can work about it with entire comfort.

The results attained with it seem to be far more easy and certain than the old style furnace. From two to four gallons of coke is quite sufficient for a single operation, and, indeed, for two dentures if the work is skillfully conducted.

No one now need object to taking up continuous gum work because of furnace labor or difficulty.

It is easier to make a piece of continuous gum work with this furnace than to make a piece of rubber work with the ordinary vulcanizer.

The introduction of this furnace makes an era in prosthetic dentistry, as distinct and important as the introduction of the dental engine or rubber dam in operative dentistry. And if it is the means of reducing, even in some small degree, the almost universal use of rubber as a base for artificial teeth, it should be hailed as a boon by every true dentist, and Dr. Tees' should be regarded as a benefactor to his profession and to the toothless.

